# Case report: Mesothelioma and BAP1 tumor predisposition syndrome: Implications for public health

**DOI:** 10.3389/fonc.2022.966063

**Published:** 2022-08-04

**Authors:** Luigi Vimercati, Domenica Cavone, Francesco Fortarezza, Maria Celeste Delfino, Romina Ficarella, Angela Gentile, Angela De Palma, Giuseppe Marulli, Luigi De Maria, Concetta Caporusso, Andrea Marzullo, Antonio d’Amati, Daniele Egidio Romano, Antonio Caputi, Stefania Sponselli, Gabriella Serio, Federica Pezzuto

**Affiliations:** ^1^ Interdisciplinary Department of Medicine, Occupational Medicine Section Ramazzini, University of Bari Aldo Moro, Bari, Italy; ^2^ Pathology Unit, Department of Medicine, School of Medicine and Surgery, University Hospital of Padova, University of Padova, Padova, Italy; ^3^ Medical Genetics Unit, Department of Human Reproductive Medicine, ASL Bari, Bari, Italy; ^4^ Thoracic Surgery Unit, Department of Emergency and Organ Transplantation, University Hospital of Bari, Bari, Italy; ^5^ Department of Emergency and Organ Transplantation (DETO), Pathological Anatomy Section, University of Bari Aldo Moro, Bari, Italy; ^6^ Department of Cardiac, Thoracic, Vascular Sciences and Public Health (DCTV), Pathology Unit, University of Padova, Padova, Italy

**Keywords:** mesothelioma, BAP1, BAP1 syndrome, familial cluster, genetic screening, public health, epidemiological surveillance

## Abstract

BRCA-1 associated protein 1 (BAP1) tumour predisposition syndrome (TPDS) is a hereditary condition characterised by germline mutation of the tumour suppressor BAP1. This disorder is associated with the development of various benign and malignant tumours, mainly involving the skin, eyes, kidneys, and mesothelium. In this article, we report the case of a man recruited through the Apulia (Southern Italy) Mesothelioma Regional Operational Centre of the National Register of Mesotheliomas, who suffered from uveal melanoma, renal cancer, and mesothelioma, and a familial cluster of BAP1 germline mutations demonstrated by molecular analyses. The family members of the proband developed multiple malignancies. As tumours arising in this context have specific peculiarities in terms of clinical behaviour, identification of this condition through appropriate genetic counselling should be considered for adequate primary, secondary, and tertiary prevention measures for offspring.

## Introduction

Malignant mesotheliomas (MMs) are relatively rare tumours, with an incidence of less than 5/100,000 per year, and MMs arise from the inner lining of the serous cavities of the pleura (93.2%), peritoneum (6.3%), pericardium (0.2%), and tunica vaginalis of the testicle (0.3%). The mean age at MM diagnosis was 70 years, with a predominance of men (M/F 2.6) (1). As the diagnosis is often made at an advanced stage, the prognosis is very poor, with a median survival of 6-12 months and 5-year survival rate of <5%. Epithelioid MM is the most common histological subtype and is associated with better survival than other subtypes, although it still lasts less than 1 year. The risk of mesothelioma is related to cumulative asbestos exposure over time, assessed according to the intensity, frequency, and duration of exposure multiplied by time in years. The Helsinki criteria (2) adopted a minimum 10-year latency interval to assign the cause of mesothelioma to asbestos. Cases with shorter latency have been reported, and there may be cases with earlier and shorter asbestos exposures that have not been recognised because of the lack of an accurate assessment of asbestos exposure. Referring to short latency, mesothelioma is now diagnosed earlier *via* cytology, and non-invasive forms have been identified, such as *in situ* mesothelioma, which may affect the possibility of early diagnosis (3).

In recent years, the incidence of mesothelioma cases without an apparent association with asbestos exposure or with lower cumulative asbestos exposure has been increasing, and recent molecular findings suggest a genetic predisposition (4, 5). Knowledge about the existence of genetic predispositions was also found in a cluster of mesothelioma cases in some families, which may be due not only to shared exposure to asbestos, but also to genetic susceptibility (6, 7).

At least 12 genes (BRCA-1 associated protein 1 (BAP1), cyclin dependent kinase inhibitor 2A-CDKN2A, Partner And Localiser Of BRCA2-PALB2, BRCA1, FA Complementation Group I-FANCI, Ataxia telangiectasia mutated-ATM, SLX4, BRCA2, FA Complementation Group C-FANCC, FA Complementation Group F-FANCF, PMS1, and Xeroderma pigmentosum, complementation group C-XPC) have been identified as involved in predisposition to MM, most of which play a role in DNA repair mechanisms. Patients carrying mutations in these genes have been shown to have an increased susceptibility to asbestos carcinogenic effects (4).

With special reference to BAP1, a broad spectrum of tumours has been associated with germline and somatic mutations in its loci, but the biological and clinical significance of these mutations is still unclear. Hereditary BAP1 tumour predisposition syndrome (TPSD) was associated with the development of a variety of benign and malignant tumours, particularly uveal melanoma, malignant mesothelioma, cutaneous melanomas, basal cell carcinomas and renal carcinoma (8).

In this article, we report a familial cluster of BAP1-TPDS, identified through a comprehensive molecular approach extended to all relatives. BAP1 syndrome and its implications for public health challenges are discussed.

## Case description

**Figure 1:**
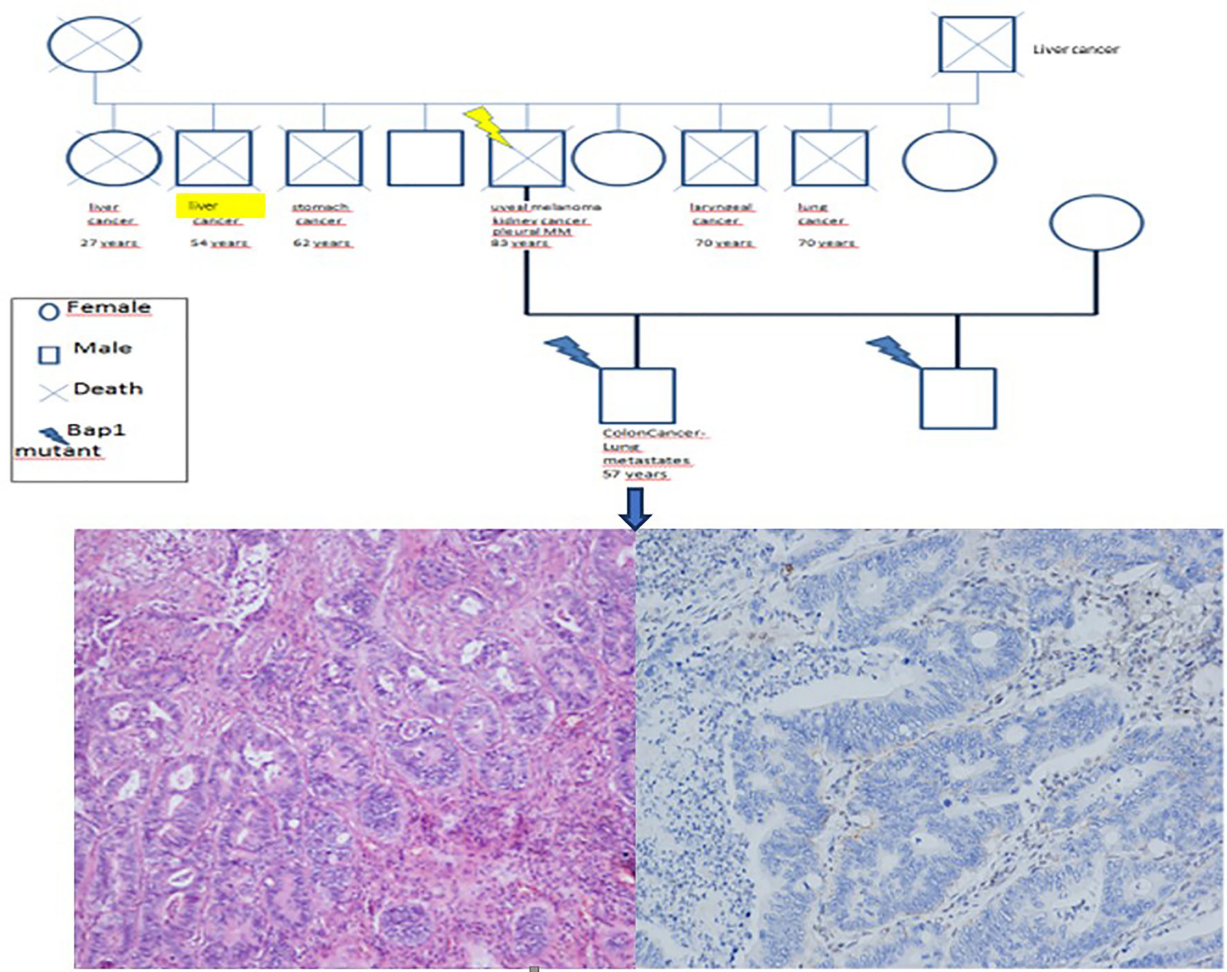
Histological review of the uveal melanoma (**A**, haematoxylin and eosin staining, 100x original magnification) and renal carcinoma (**C**, haematoxylin and eosin staining, 100x original magnification). In both tumours, BAP1 was negative by immunohistochemistry (**B, D**, immunohistochemistry, 200X).

In December 2020, an 83-year-old man, who was a nonsmoker, was admitted to our hospital with worsening pharyngodynia. Chest radiography revealed massive right pleural effusion that was treated with pleural drainage and pleural talcing. In 1992, the patient was surgically treated for uveal melanoma of the right eye (**Figure 1**), which recurred in 2012. Diffuse polyposis of the colon was discovered during coloscopy in 2019. Moreover, in the same year, he underwent nephrectomy for right clear cell renal cancer (**Figure 1**). Samples from all previous tumours were available for histological analysis.

**Figure 2:**
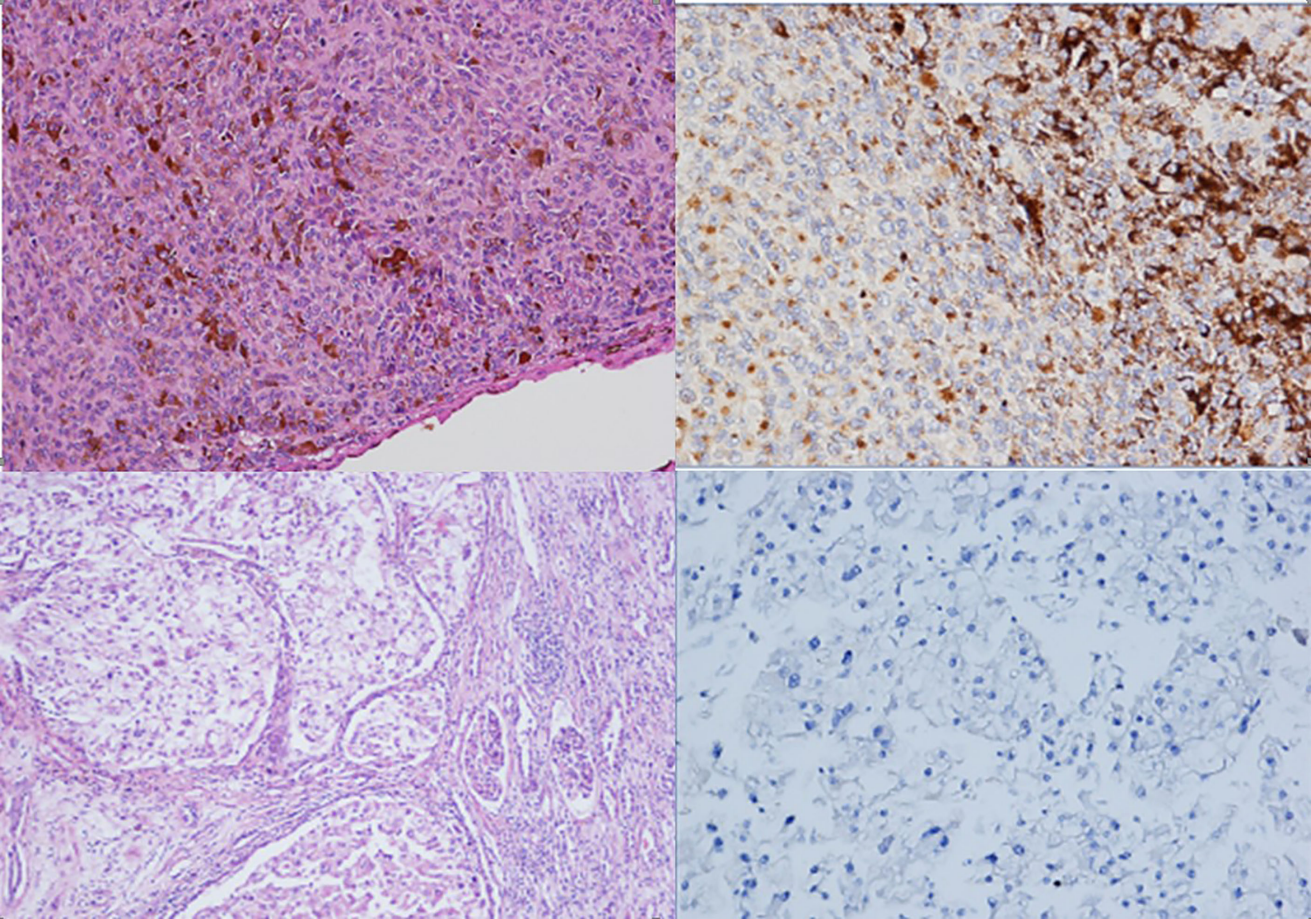
Histological examination of the pleural samples, showing a neoplastic proliferation composed of both epithelioid and spindle cells (**A**, haematoxylin and eosin staining, 100x) that was immunoreactive for calretinin (**B**, immunohistochemistry, 200x) and negative for BAP1 (**C**, immunohistochemistry, 100x), with CDKN2A/p16 deletion visualized *via* FISH analysis **(D)**.

A pleurectomy was performed as per the operating procedure in February 2021. Histological examination showed proliferation of epithelioid malignant cells with solid and trabecular patterns (70%), together with spindle cells arranged in bundles (30%). According to recent guidelines (9, 10), an examination using a panel of antibodies was performed. Neoplastic cells were positive for cytokeratin AE1/AE3 ((Dako, Glostrup, Denmark), calretinin (Zymed, S. Francisco, CA, USA), HBME1 (Dako), and WT1 (Novocastra) and negative for PAX8 (Invitrogen), S100 (Dako), TTF1 (Dako), and BAP1 (Santa Cruz Biotechnology Inc., clone C4). FISH analysis was performed as previously described (11) and revealed the presence of a deletion in the 9p21 locus of the CDKN2A gene in > 20% of neoplastic cells (**Figure 2**). A definitive diagnosis of biphasic mesothelioma was made. The patient died a few days after hospitalisation due to SARS-CoV-2 infection. The case was registered in the mesothelioma register, and the patients’ sons were interviewed using a semi-structured questionnaire according to the guidelines of the National Mesothelioma Registry (12) to build a comprehensive anamnesis to identify any potential work or environmental exposure, residential history, nonwork habits (free time or at-risk activities), and any exposure in the family environment (work activities of parents, siblings, and spouse).

The patient had both environmental asbestos exposure (military service carried out approximately 60 years earlier in Casale Monferrato, a town well known for environmental pollution by asbestos due to the presence of Eternit, a company for the production of asbestos cement) and professional exposure to asbestos (as an electrical maintenance technician in a steel mill for 22 years).

Residential and non-working history was negative for exposure to asbestos, and the use of talcum powder for hygienic purposes was positive in the anamnesis.

**Figure 3:**
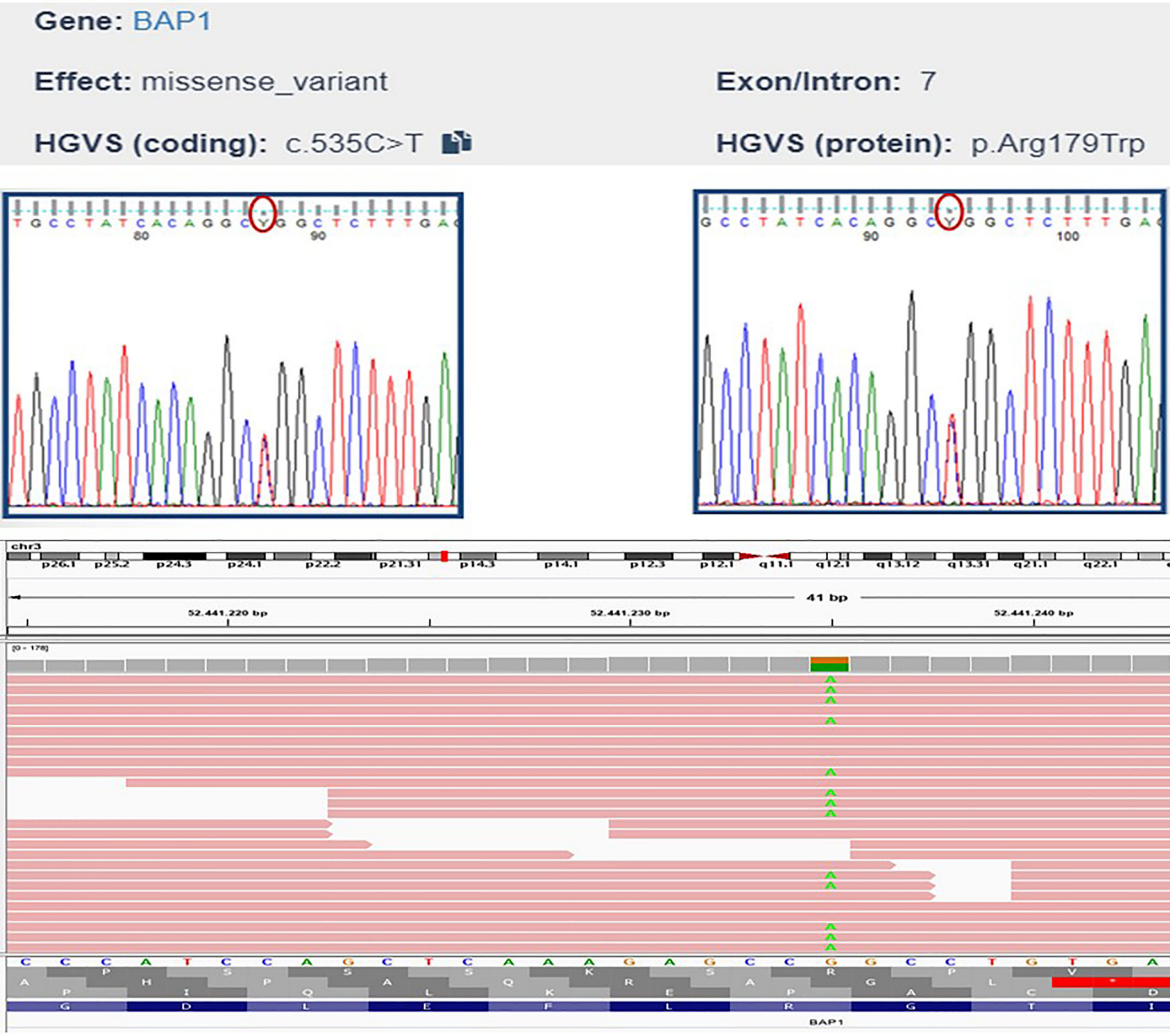
Familial history of neoplasia in the patient’s relatives **(A)**. The histological review of the son’s colorectal carcinoma sample showed adenocarcinoma (**B**, haematoxylin and eosin staining, 100x original magnification) that was negative for BAP1 expression (**C**, immunohistochemistry, 100x original magnification).

Moreover, a 36-year-old former smoker, one of the sons, was affected by moderately differentiated colorectal adenocarcinoma (stage T4N1bM0) treated with hemicolectomy and 12 cycles of chemotherapy. Colorectal cancer recurred in 2021 with lung metastases (**Figure 3**). In his son’s work and residential history, exposure to asbestos and other carcinogens was negative.

Interestingly, reconstruction of the entire family history showed a high incidence of oncological pathologies among all relatives. Specifically, the patient’s father, sister, and brother died of liver cancer, and two other siblings died of laryngeal and lung cancer (**Figure 3**).

When BAP1-TPDS was suspected, germline mutations of BAP1 were identified in the peripheral blood samples of the patient and his sons after obtaining written informed consent.

## Genetic analyses

**Figure 4:**
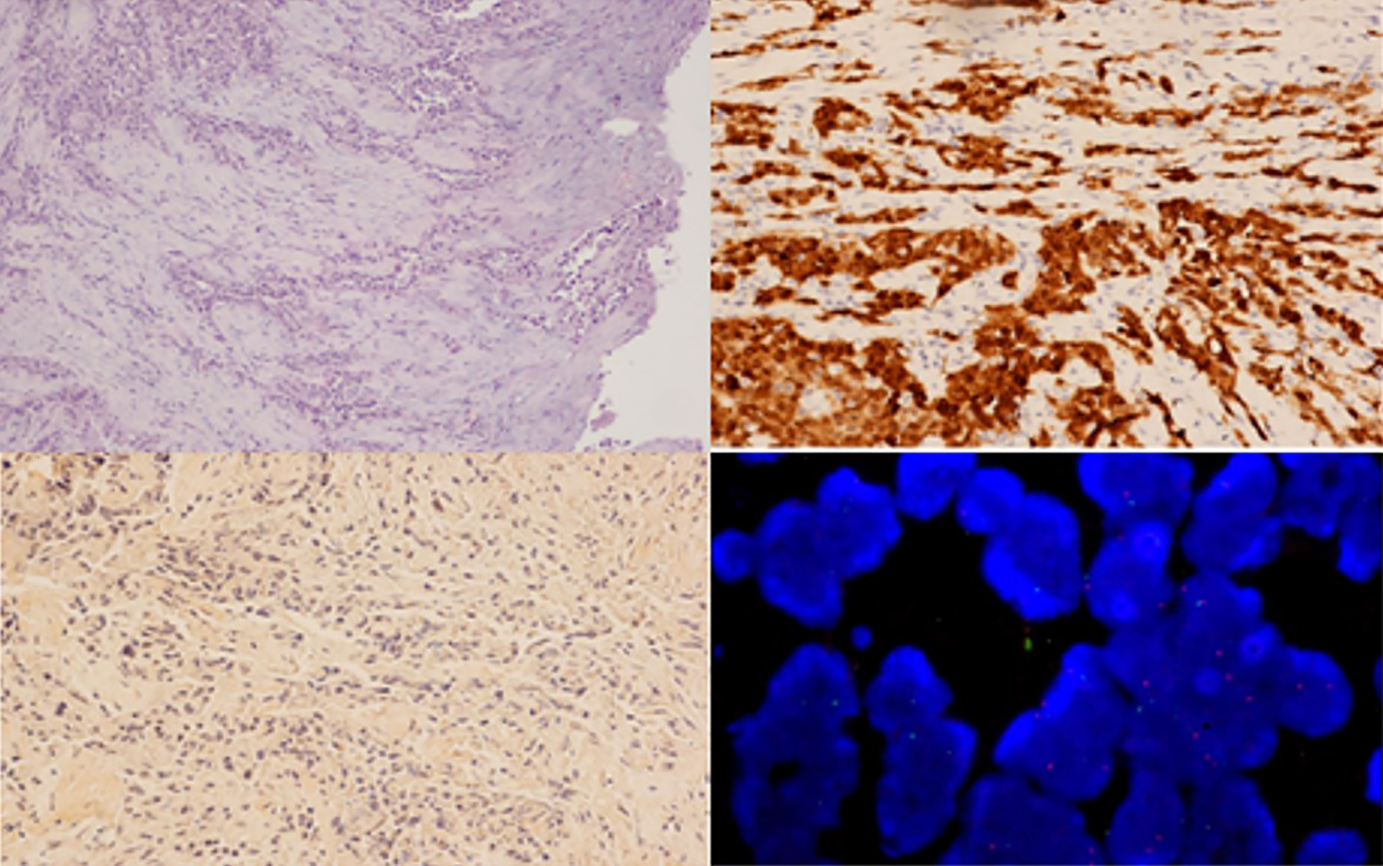
Sanger sequencing electropherogram and IGV alignment of the BAP1 variant c.535C>T.

Genomic DNA was extracted using standard protocols. The resulting DNA sample was then used to construct DNA libraries using Illumina DNA Prep with Enrichment and TruSight One Sequencing Panels (Illumina^®^, San Diego, CA, USA), which provides comprehensive coverage of > 4800 disease-associated genes to verify the presence of other tumour susceptibility genes. Sequencing was performed on an Illumina^®^ MiSeq desktop sequencer (MiSeq Reagent Kit v3, Illumina^®^, San Diego, CA, USA), and data were analysed using Illumina^®^ Variant Interpreter Software (v2.14.0.4). The test did not detect duplications or deletions of one or more exons or the entire gene and a low percentage of mosaicisms and epimutations. The analysis revealed a heterozygous missense mutation in exon 7 of BAP1, c.535C>T (Chr3:52441235 on Assembly GRCh37, p.Arg179Trp). The variant was validated using Sanger sequencing (**Figure 4**). This BAP1 variant was reported in the ClinVar database as a variant of uncertain significance (VOUS), and in silico prediction classified the variant as being of uncertain significance based on the following ACMG criteria: likely pathogenic based on computational prediction tools (PP3 supporting criteria), and variant not found in the GnomAD Exomes database (PM2 supporting criteria). Unfortunately our analysis did not detect any other VUS or germline mutations in any member of the family group we analysed. However this BAP1 variant was located in the functional peptidase domain of the protein (Pfam protein family database prediction). Considering the intrinsic characteristics of the method (regions rich in GC and homopolyneric sequences), some mutations could have escaped mutational analysis.

## Discussion

In this case series, we report a familial cluster of BAP1-TPDS in family members who developed multiple malignancies with BAP1 germline mutations, as demonstrated by molecular analyses. To the best of our knowledge, this is the ninth description of this syndrome in Italy in the literature (13). The multidisciplinary approach to the patient and the accuracy of the reconstruction of the whole patient and family history allowed us to further investigate an apparently usual MM. BAP1 loss was demonstrated in all tumours by immunohistochemistry (IHC) and germline mutations were detected by molecular tests.

### 1 BAP1 mutation and mesothelioma

BRCA1-associated protein 1 (BAP1), encoded by the gene (MIM # 603089) in OMIM (Online Mendelian Inheritance in Man (OMIM), has a tumour suppressor function and is a 729 amino acid carboxy-terminal ubiquitin hydrolase that plays a role in cell cycle progression, DNA damage response (DDR), histone modification, apoptosis, DNA replication, and DNA repair. BAP1 is found in 3p21.3, a region of chromosome 3 in which deletions commonly occur, and is located on the short arm (p) of chromosome 3 at position 21.1 (14, 15). Han et al. (16) summarised and divided the metabolic roles of BAP1 into four main categories: i) nutrient homeostasis, ii) glycolytic alterations, iii) calcium dysregulation, and iv) metabolic stress management (16).

BAP1 functions as a true tumour suppressor gene with biallelically inactivating mutations or deletions (17, 18). To lose BAP1 tumour suppressor function in a cell, the normal wild-type allele must acquire a somatic mutation (17, 18). Heterozygous germline variants in this gene may favour carcinogenesis; therefore, people with BAP1 mutations carry a high risk for cancer, especially cancers that are caused by environmental carcinogens. Thus, gene–environment interactions are suspected to play an important role in cancer susceptibility of BAP1 mutation carriers (7, 14).

Several alterations in BAP1 have been described, including deletions of the coding sequence, base substitutions leading to nonsense and missense mutations, focal deletions, frameshift mutations, deletions, and large chromosomal insertions or splice site mutations. These somatic variants are of different types and include protein-truncating variants (PTVs), large deletions, or chromosomal loss. BAP1 Somatic inactivation is a common event in “sporadic” tumours (14, 17). BAP1 deficiency leads to accumulation of DNA damage, which increases the chance of neoplastic proliferation in patients with both sporadic and inherited deficiencies of BAP1. BAP1 mutations favour the development of different tumour phenotypes and have been identified in a wide range of solid tumour types, including malignant mesothelioma (MM) (16, 17).

Regarding mesothelioma, Hammady (18) reported a possible genetic susceptibility factor for MM in the Cappadocian region of Turkey, in which some families were particularly susceptible to developing mesothelioma due to the presence of germline BAP1 mutations transmitted according to Mendelian law. Both germline and sporadic BAP1 mutations have been shown to play a role in MM, and these patients have a family history of MM with limited asbestos exposure (19). BAP1 loss occurs in both sporadic and familial MM, either pleural or peritoneal, independent of ethnic background, sex, age, or other clinical characteristics. MM patients with germline BAP1 mutations have an overall 7-fold increased long-term survival; moreover, they have been associated with a younger age at MM onset (20). The association between BAP1 germline mutations and asbestos exposure in MM remains unknown. BAP1 germline mutations in MM are related to a favourable prognosis (16) and higher sensitivity to chemotherapy, but are also a negative predictor of response to chemotherapy and could possibly be used as a companion biomarker for treatment decision-making. Moreover, BAP1 germline mutations could be used as predictive biomarkers of immunotherapy response (21), that is, to predict elevated responsiveness to immunotherapy for future precision cancer medicine (5, 22), and in screening to identify subjects and their family members at risk of developing cancer (23).

The expression of BAP1 visualised by IHC represents a biomarker with excellent clinical utility for the diagnosis of malignant mesothelioma and is indicative of prognosis. BAP1 loss has shown 100% specificity for distinguishing mesothelial proliferation, for example, benign (with positive nuclear staining) from malignant (with negative nuclear staining) mesothelial cells (21). BAP1 IHC is an inexpensive and quick way to screen tumours with BAP1 mutations since paraffin-embedded tumour tissue is readily available. Loss of nuclear BAP1 staining by immunohistochemistry is thought to be an accessible and reliable test for BAP1 mutations. Tumours that present with loss of nuclear BAP1 expression may then undergo subsequent confirmatory sequencing. Bap1 IHC negativity has been previously described in epithelioid malignant pleural mesothelioma (MPM) (70%) and biphasic (60%) types but has also been observed in sarcomatoid and desmoplastic mesothelioma, albeit less frequently (15%), so it may serve as a predictive biomarker (14).

Recently, whole-exome sequencing confirmed that mesothelioma *in situ* development is associated with BAP1 somatic mutations/deletions, suggesting that BAP1 mutations/deletions represent a very early event in the development of malignant mesothelioma (3). Recently, new diagnostic tools, such as radiogenomics, have helped identify the potential correlation between imaging findings and tumour genotype. CT-based 3D radiomics signatures have potential as noninvasive markers for the prediction of BAP1 mutation status in patients with mesothelioma MPM as well as for differential diagnosis and prediction of response to therapy and prognosis (24).

Because BAP1 regulates DNA repair in patients with damage caused by asbestos, ultraviolet light, radiation, or chemotherapy, carriers of this mutation are less able to repair DNA damage induced by carcinogens, leading to the accumulation of mutations associated with carcinogenesis (14, 23, 25). Germline mutations in BAP1 increase susceptibility to low amounts of asbestos and could increase the risk of developing mesothelioma among carriers of this mutation, with an exposure dose considered harmless for the general population. Recently, some studies on the tumour immune microenvironment and genetic alterations in mesothelioma have speculated that excessive phagocytosis of asbestos fibres by macrophages provides “a mutagenic microenvironment” around mesothelial cells and induces BAP1 mutation, which is related to the suppression of mesothelial cell death and the accumulation of additional mutations involved in mesothelioma carcinogenesis, all of which are characterised as a highly inflammatory TME (tumour microenvironment) that may be important for immunotherapeutic approaches (26). Recently, Panou (4) reported several single nucleotide polymorphisms (SNPs) and the presence of a missense variant in exon 11 of BAP1 in exposed patients, which may promote MM tumourigenesis as a result of asbestos–gene interaction and prolonged survival. Indeed, the gene–environment (gene-asbestos) interaction (GXE) is suspected to play an important role in cancer susceptibility for BAP1 mutation carriers, which could be used for cancer prevention in high-risk individuals as well as for targeted therapies (27). Combined genetic and environmental analyses may be useful for studying the accumulation of spontaneous somatic mutations due to exposure to carcinogenic substances such as asbestos, and the presence of inherited mutations (germline) occurring in genes that are critical for DNA repair and/or homologous recombination (HR) may have a synergistic effect (28). The discovery of a susceptibility gene may be a useful tool for defining high-risk versus low-risk groups for screening asbestos-exposed individuals with novel noninvasive biomarkers.

BAP1 may also be a possible therapeutic target for precision medicine and prognostic biomarkers, being frequently altered in MM (29) together with other targets reported thus far (30, 31). The 15th conference of the International Mesothelioma Interest Group (iMig), held in May 2021, demonstrated the usefulness of pleural effusions for the early diagnosis of MM. Cytological analysis of gene expression profiles in samples discriminated reactive hyperplasia from epithelioid mesothelioma, and prognostic biomarkers, such as alterations in BAP1 (IHC) and CDKN2A (FISH), were detectable almost two years prior to diagnosis (32).

### 2 BAP1-TPDS and familial clusters

BAP1 tumour predisposition syndrome (BAP1-TPDS) is inherited in an autosomal-dominant manner, with individuals carrying a heterozygous BAP1 mutation being at high risk for various tumours (MIM #614327 in Online Mendelian Inheritance in Man (OMIM)), and was first described in the early 2000s (18). BAP1 cancer syndrome includes mesothelioma (33), uveal melanoma (34), cutaneous melanoma, and possibly other malignant tumours. BAP1-TPDS is associated with an increased risk of a specific skin lesion, BAP1-inactivated melanocytic tumour (BIMT) (34), cutaneous melanoma (CM) (34), renal cell carcinoma (RCC) (35), and basal cell carcinoma (BCC) (36). The occurrence of hepatocellular carcinoma, cholangiocarcinoma (37), and meningioma (8) has also been described. The associations between BAP1-TPDS and breast cancer, neuroendocrine carcinoma, non-small cell lung adenocarcinoma (8), colorectal cancer (38), thyroid cancer (39), urinary bladder cancer, thymic epithelial tumours (40), paraganglioma (41), meningioma (42), oesophageal cancer, cholangiocarcinoma (43), and endometrial carcinoma (44) are currently under evaluation. Affected individuals can have more than one type of primary cancer. A common trait is that the median age of the onset of these tumours is younger in patients with BAP1-TPDS than in the general population.

MM is the second most common cancer in patients with BAP1-TPDS, accounting for 17% of non-probands and 25% of probands with *BAP1* null variants (13). Even when the family history is not suggestive of BAP1-TPDS, genetic testing for *BAP1* variants could be considered if the patient has ≥2 BAP1-TPDS core tumours (33). If a parent of the proband carried the BAP1 pathogenic variant identified in the proband, the risk for siblings inheriting the variant was 50%. However, penetrance appears to be incomplete and the types of BAP1-related cancers can vary among different members of the same family (25, 45). Moreover, patients with mesothelioma had improved survival with respect to patients with sporadic mesothelioma, independent of patient age at presentation (46). Similar to mesotheliomas caused by environmental exposure, those linked to inherited germline mutations occur with an M:F ratio close to 1:1, are morphologically almost exclusively of the epithelioid type, and are well differentiated. Familial BAP1-related MM frequently affects women (19). Familial BAP1-related MMs can be multiple, often occur in association with other synchronous or metachronous neoplasms, and are more commonly located in the peritoneum than sporadic MMs (20).

Many studies have evaluated blood-related cases of mesothelioma involving several members of the same family (6, 7). Walpole (13) reported 181 different families worldwide carrying BAP1 mutations; however, this syndrome remains under-recognized and under-reported (20, 47).

Early detection screening for germline BAP1 mutations is necessary to identify individuals and their family members who carry BAP1 germline mutations and are at high risk of developing associated cancers (7, 16, 22, 23); Thus, genetic counselling and clinical management are important for carriers of germline BAP1 mutations and to reduce exposure to even minimal sources of carcinogens. Carriers should be advised to limit their exposure to potential carcinogens, such as sunlight, avoid asbestos, and avoid arc welding and tobacco exposure. Moreover, these individuals should limit their exposure to diagnostic and therapeutic ionising radiation and implement preventative measures to limit environmental carcinogenic exposure. Early detection of this mutation has been shown to increase the overall survival in these predisposed individuals due to close screening and monitoring, as well as more therapeutic options for early stages of cancer and in view of the future availability of promising targeted therapies for BAP1-mutated tumours (6, 15).

The diagnosis of bap1 syndrome is based on the genetic examination indicating that at least two BAP1 TPDS-associated tumours were present in the patient’s clinical history, if at least one primary bap1 TPDS tumour is reported in the clinical history of first- and second-generation relatives, and if the age of onset reported is young (46). If a patient (or a family member) is clinically suspected of having a BAP1 germline mutation on the basis of their clinical phenotype (and/or familial pedigree), following appropriate genetic counselling, it is recommended to test BAP1 with IHC, which is routinely available, and for blood-related family members to undergo direct (Sanger) sequencing using blood-derived DNA. Sanger sequencing should also be performed for patients with uncertain IHC results. It is important to collect detailed medical histories of the patients’ blood-related family members, including their occupational and oncological histories.

To date, many studies have shared prevention protocols for this syndrome (25, 45, 46, 48). A multidisciplinary approach was suggested, recommending active surveillance programs, with annual screening that should preferably be performed in one day, where patients are screened for BAP1-TPDS-associated malignancies by the internist, oncologist, pulmonologist, ophthalmologist, dermatologist, or radiologist, starting from 11 to 16 years of age for uveal melanoma or from 30 years of age for other pathologies with a six-month, annual, or biennial frequency. Among the most recent revisions, Pilarski (25). updated the surveillance protocols and emphasised the importance of both pertinent follow-up in compliance with the principles of a good screening test (25) and psychological support to reduce the risk of screening burnout and loss to follow-up, as participants in a BAP1 screening program may experience negative impacts such as early discovery of tumours without adequate treatment options (25, 49). There is a general consensus on the need for genetic counselling to provide individuals and families with information on the nature and mode of inheritance, and the implications of genetic disorders to help them make informed medical and personal decisions for both relatives and siblings, including the offspring of a patient who is positive for a Bap1 germline mutation. Once a germline BAP1 pathogenic variant has been identified in an affected family member, prenatal genetic testing for BAP1-TPDS is possible (25). Genetic surveillance of patients with BAP1-TPDS opens up problems with legal and insurance implications for the healthcare system. Testing in younger and older individuals may lead to implications with respect to the ethical principles of autonomy, justice, beneficence, and non-maleficence, and represents a new challenge in public health and preventive medicine. An ethical problem of possible discrimination and effective prevention at the same time may be related to genetic testing for BAP1 mutations in exposed worker cohorts, which will help identify genetically susceptible individuals for appropriate prevention measures, considering that carriers of a germline BAP1 pathogenic variant tend to develop BAP1-TPDS-associated malignancies at a younger age (7). From the same preventive perspective, patients who present with clinical indicators denoting heritability (a familial history of mesothelioma or other cancers at a young age [ ≤ 50 years]) should undergo genetic testing by targeted next-generation sequencing (NGS) using a gene panel covering all DNA repair and tumour suppressor genes to test for cancer inheritability. Regarding the public health problems posed by this syndrome, in a health economic study, Wallpole (48). proved that active surveillance in genomic medicine applications, as germline testing is becoming more common and less expensive, could improve survival and be cost-effective for the healthcare system. Using mathematical modelling and computer simulations, the authors found that surveillance decreased BAP1 cancer-related death mortality from 50% to 35%, increased life expectancy by 4.9 years and avoided high healthcare costs associated with advanced cancers (48).

## Conclusions

Tumours arising in the context of BAP1-TPDS have specific peculiarities in terms of clinical behaviour, and it is not surprising that the identification of family clusters is not always easy. The family history of some individuals diagnosed with BAP1-TPDS may appear negative due to the non-recognition of the disorder in other family members, reduced penetrance, premature death of the parent before the onset of symptoms, or late onset of the disease in the affected person.

In the cluster described here a strong point is that we were able to reconstruct the paternal branch oncological family history, in agreement with previous studies that reported how most people diagnosed with BAP1-TPDS have an affected parent (6, 7). However a limitation is that in the case of the siblings and father of our patient, genetic investigations were never carried out; therefore, the possibility of gene mutations remains unknown. Given the concomitance of pathologies that are part of BAP1-TPDS, strong family oncological positivity, and the presence of an investigated and ascertained mutation in our patient and his son, it is assumed that the other members of our patient’s family could also be carriers of this susceptibility. It should be noted that immunohistochemical examination of our patient’s tumour revealed negativity for BAP-1, but showed strong positivity for CDK2A. Furthermore, an affected parent may have BAP1-related tumours that differ from those of the proband. In the cases described here, the tumours of the father were in fact different from those of the affected son.

Other strong points are the accurate occupational history obtained by the occupational doctor, supported by genetic investigations and anatomopathological data, allowed us to identify two subjects at risk of contracting uveal melanoma, skin tumours, kidney tumours, malignant mesothelioma, or other related tumours due to the BAP1 mutation.

It was thus possible to start these patients on a path of health surveillance, based on the most recent indications of the scientific community, to perform effective prevention and counselling and possibly to make an early disease diagnosis, subject them to any innovative therapeutic treatments to improve their quality of life and extend their survival.

The identification of genetic mutations in family members affected by MM, and as in our case, by other pathologies included in BPA1-TPDS, must alert doctors to implement genetic counselling, screening measures, and therapeutic measures for oncological pathologies (47). The importance of detecting families affected by this type of mutation must be evaluated with a view to primary prevention, since it is necessary to protect these families from exposure to various carcinogens, both environmental and occupational, to prevent the development of malignant diseases.

Here, we report the case of an individual in whom three distinct malignant tumours were diagnosed approximately 10 years apart. The detection of BAP1 loss by IHC has been recognised as highly specific for MM, although the sensitivity of BAP1 loss detection is relatively low, approximately 50% for epithelioid histology, and even lower for sarcomatoid histology (50). Our patient was predominantly affected by sarcomatoid biphasic MM of the pleura, which could explain the negativity of IHC for BAP1. Missense mutations in the active site of the ubiquitin hydrolase domain (Arg179Tp) were identified in the patient’s son. Similar missense mutations have been described in the literature for uveal melanomas (p.Ser172Arg) and biphasic pleural mesotheliomas (p.Tyr173Ser), both of which our patient had (6, 17).

This multidisciplinary approach included the patient’s sons in specific clinical and instrumental surveillance protocols as per the guidelines in the literature (25).

Regarding the intestinal adenocarcinoma of our patient’s son, BAP1 downregulation was associated with decreased CRC survival (38). The literature shows that some families carrying missense variants can exhibit BAP1-TPDS-associated tumours, regardless of the uncertainty surrounding the functional impact of germline missense substitutions in BAP1, to date classified as variants of unknown significance (VUS). Consideration of the tumour spectrum within a family of missense VUS carriers can help to evaluate variant classification and pathogenicity (13, 51).

Challenges in the genetic surveillance of Bap1 mutation carriers, that is, precision prevention, are related to limited data on cancer risks for unaffected carriers and a lack of evidence-based surveillance benefits. Other critical points in screening are the cost of public health, multidisciplinary surveillance, insurance implications, the risk of genetic discrimination, and the need for psychological support for these subjects (45).

As part of a novel multidisciplinary approach, physicians, including occupational doctors involved in the epidemiological and health surveillance of subjects exposed to asbestos, and all other specialists involved in diagnostics should be aware of the histological features of TPDS tumours, and once detected in affected individuals, the identification of families carrying germline BAP1 mutations is mandatory to start appropriate surveillance programmes and guarantee the best clinical management for these patients (20). Three steps can be envisaged: (1) clinical assessment and genetic counselling, (2) pathological and molecular diagnosis, and (3) risk assessment, early prevention, and therapies that can impact outcomes and survival (23).

In conclusion, epidemiological surveillance carried out through a mesothelioma registry may be a useful tool to promote a multidisciplinary approach involving occupational doctors, pathologists, radiologists, surgeons, oncologists, and geneticists in the monitoring of patients with an undiagnosed mutation. The consequences of epidemiological surveillance in terms of public health allow primary, secondary, and tertiary prevention for family carriers of the same mutation, with a possible reduction in both human life and economic costs for the community.

## Data availability statement

The datasets presented in this study can be found in online repositories. The names of the repository/repositories and accession number(s) can be found in the article/supplementary material.

## Ethics statement

The studies involving human participants were reviewed and approved by Ethics Committee of the University Hospital Polyclinic of Bari, Bari, Italy (number 5062, date June 22, 2016). The patients/participants provided their written informed consent to participate in this study. Written informed consent was obtained from the patients for publication of this paper.

## Author contributions

Conceptualisation, LV, DC, GS. Methodology, DC, GS, AP, GM. software, DC, FF. Validation, FF, AM, FP. Formal analysis, AG, RF. investigation, LM, CC, Ad’A, DR, AC, SS. Data curation, DC, GS. Writing—original draft preparation, DC, MD. Writing—review and editing, DC, FF, LV, GS. Supervision, LV, GS. All authors contributed to the article and have seen and approved the submitted version.

## Conflict of interest

The authors declare that the research was conducted in the absence of any commercial or financial relationships that could be construed as a potential conflict of interest.

## Publisher’s note

All claims expressed in this article are solely those of the authors and do not necessarily represent those of their affiliated organizations, or those of the publisher, the editors and the reviewers. Any product that may be evaluated in this article, or claim that may be made by its manufacturer, is not guaranteed or endorsed by the publisher.

## References

[1] . Available at: https://www.inail.it/cs/internet/docs/alg-pubbl-il-registro-nazionale-mesoteliomi-settimo-rapporto.pdf (Accessed 5 May 2022).

[2] Tossavainen A . Asbestos, asbestosis, and cancer: The Helsinki criteria for diagnosis and attribution. Scand J Work Environ Health (1997) 23:311–6. doi: 10.5271/sjweh.226 9322824

[3] Klebe S . Progression of mesothelioma in situ to invasive disease 4 years and 10 months after initial diagnosis. Pathology (2022),54(3): 384–6. doi: 10.1016/j.pathol.2021.06.124 34565603

[4] Panou V Røe OD . Inherited genetic mutations and polymorphisms in malignant mesothelioma: A comprehensive review. Int J Mol Sci (2020) 21(12):4327. doi: 10.3390/ijms21124327 PMC7352726 32560575

[5] Sculco M La Vecchia M Aspesi A Pinton G Clavenna MG Casalone E . Malignant pleural mesothelioma: Germline variants in DNA repair genes may steer tailored treatment. Eur J Cancer (2022) 163:44–54. doi: 10.1016/j.ejca.2021.12.023 35032816

[6] Ohar JA Cheung M Talarchek J Howard SE Howard TD Hesdorffer M . Germline BAP1 mutational landscape of asbestos-exposed malignant mesothelioma patients with family history of cancer. Cancer Res (2016) 76:206e15. doi: 10.1158/0008-5472.CAN-15-0295 26719535 PMC4715907

[7] Chau C van Doorn R van Poppelen NM van der Stoep N Mensenkamp AR Sijmons RH . Families with BAP1-tumor predisposition syndrome in the Netherlands: Path to identification and a proposal for genetic screening guidelines. Cancers (Basel) (2019) 11(8):1114. doi: 10.3390/cancers11081114 PMC6721807 31382694

[8] Abdel-Rahman MH Pilarski R Cebulla CM Massengill JB Christopher BN Boru G . Germline BAP1 mutation predisposes to uveal melanoma, lung adenocarcinoma, meningioma, and other cancers. J Med Genet (2011) 48(12):856–9. doi: 10.1136/jmedgenet-2011-100156 PMC3825099 21941004

[9] WHO Classification of Tumours . Thoracic tumour. 5th ed. Lyon, France: WHO (2021).

[10] Pezzuto F Serio G Fortarezza F Scattone A Caporusso C Punzi A . Prognostic value of Ki67 percentage, WT-1 expression and p16/CDKN2A deletion in diffuse malignant peritoneal mesothelioma: A single-centre cohort study. Diagnostics (Basel) (2020) 10(6):386. doi: 10.3390/diagnostics10060386 PMC7345555 32526924

[11] Husain AN Colby TV Ordóñez NG Allen TC Attanoos RL Beasley MB . Guidelines for pathologic diagnosis of malignant mesothelioma 2017 update of the consensus statement from the international mesothelioma interest group. Arch Pathol Lab Med (2018) 142(1):89–108. doi: 10.5858/arpa.2017-0124-RA 28686500

[12] . Available at: https://www.inail.it/cs/internet/docs/all-linee-guida-renam.pdf?section=attivita (Accessed 5 May 2022).

[13] Walpole S Pritchard AL Cebulla CM Pilarski R Stautberg M Davidorf FH . Comprehensive study of the clinical phenotype of germline BAP1 variant-carrying families worldwide. J Natl Cancer Inst (2018) 110(12):1328–41. doi: 10.1093/jnci/djy171 PMC6292796 30517737

[14] Carbone M Harbour JW Brugarolas J Bononi A Pagano I Dey A . Biological mechanisms and clinical significance of *BAP1* mutations in human cancer. Cancer Discov (2020) 10(8):1103–20. doi: 10.1158/2159-8290.CD-19-1220 PMC8006752 32690542

[15] Gupta S Erickson LA Lohse CM Shen W Pitel BA Knight SM . Assessment of risk of hereditary predisposition in patients with melanoma and/or mesothelioma and renal neoplasia. JAMA Netw Open (2021) 4(11):e2132615. doi: 10.1001/jamanetworkopen.2021.32615 34767027 PMC8590170

[16] Han A Purwin TJ Aplin AE . Roles of the BAP1 tumor suppressor in cell metabolism. Cancer Res (2021) 81(11):2807–14. doi: 10.1158/0008-5472.CAN-20-3430 PMC8178170 33446574

[17] Murali R Wiesner T Scolyer RA . Tumours associated with BAP1 mutations. Pathology (2013) 45(2):116–26. doi: 10.1097/PAT.0b013e32835d0efb 23277170

[18] Roushdy-Hammady I Siegel J Emri S Testa JR Carbone M . Genetic-susceptibility factor and malignant mesothelioma in the cappadocian region of Turkey. Lancet (2001) 357(9254):444–5. doi: 10.1016/S0140-6736(00)04013-7 11273069

[19] Panou V Gadiraju M Wolin A Weipert CM Skarda E Husain AN . Frequency of germline mutations in cancer susceptibility genes in malignant mesothelioma. J Clin Oncol (2018) 36(28):2863–71. doi: 10.1200/JCO.2018.78.5204 PMC6804864 30113886

[20] Pagliuca F Zito Marino F Morgillo F Della Corte C Santini M Vicidomini G . Inherited predisposition to malignant mesothelioma: Germline BAP1 mutations and beyond. Eur Rev Med Pharmacol Sci (2021) 25(12):4236–46. doi: 10.26355/eurrev_202106_26129 34227091

[21] Oehl K Vrugt B Wagner U Kirschner MB Meerang M Weder W . Alterations in *BAP1* are associated with cisplatin resistance through inhibition of apoptosis in malignant pleural mesothelioma. Clin Cancer Res (2021) 27(8):2277–91. doi: 10.1158/1078-0432.CCR-20-4037 33547197

[22] Carbone M Amelio I Affar EB Brugarolas J Cannon-Albright LA Cantley LC . Consensus report of the 8 and 9th weinman symposia on gene x environment interaction in carcinogenesis: Novel opportunities for precision medicine. Cell Death Differ (2018) 25(11):1885–904. doi: 10.1038/s41418-018-0213-5 PMC6219489 30323273

[23] Kittaneh M Berkelhammer C . Detecting germline BAP1 mutations in patients with peritoneal mesothelioma: Benefits to patient and family members. J Transl Med (2018) 16(1):194. doi: 10.1186/s12967-018-1559-7 30001711 PMC6044070

[24] Xie XJ Liu SY Chen JY Zhao Y Jiang J Wu L . Development of unenhanced CT-based imaging signature for BAP1 mutation status prediction in malignant pleural mesothelioma: Consideration of 2D and 3D segmentation. Lung Cancer (2021) 157:30–9. doi: 10.1016/j.lungcan.2021.04.023 34052706

[25] Pilarski R Carlo MI Cebulla C Abdel-Rahman M . BAP1 tumor predisposition syndrome. In: Adam MP Ardinger HH Pagon RA Wallace SE Bean LJH Gripp KW Mirzaa GM Amemiya A , editors. GeneReviews. Seattle (WA: University of Washington, Seattle (2016). p. 1993–2022. 27748099

[26] Hiltbrunner S Mannarino L Kirschner MB Opitz I Rigutto A Laure A . Tumor immune microenvironment and genetic alterations in mesothelioma. Front Oncol (2021) 11:660039. doi: 10.3389/fonc.2021.660039 34249695 PMC8261295

[27] Carbone M Arron ST Beutler B Bononi A Cavenee W Cleaver JE . Tumour predisposition and cancer syndromes as models to study gene-environment interactions. Nat Rev Cancer (2020) 20(9):533–49. doi: 10.1038/s41568-020-0265-y PMC8104546 32472073

[28] Yoshikawa Y Kuribayashi K Minami T Ohmuraya M Kijima T . Epigenetic alterations and biomarkers for immune checkpoint inhibitors-current standards and future perspectives in malignant pleural mesothelioma treatment. Front Oncol (2020) 10:554570. doi: 10.3389/fonc.2020.554570 33381446 PMC7767988

[29] Morani F Bisceglia L Rosini G Mutti L Melaiu O Landi S . Identification of overexpressed genes in malignant pleural mesothelioma. Int J Mol Sci (2021) 22(5):2738. doi: 10.3390/ijms22052738 33800494 PMC7962966

[30] Serio G Pezzuto F Marzullo A Scattone A Cavone D Punzi A . Peritoneal mesothelioma with residential asbestos exposure. Report of a case with long survival (Seventeen years) analyzed by cgh-array. Int J Mol Sci (2017) 18(8):1818. doi: 10.3390/ijms18081818 PMC5578204 28829357

[31] Serio G Vimercati L Pennella A Gentile M Cavone D Buonadonna AL . Genomic changes of chromosomes 8p23.1 and 1q21: Novel mutations in malignant mesothelioma. Lung Cancer (2018) 126:106–11. doi: 10.1016/j.lungcan.2018.10.012 30527173

[32] Klebe S Galateau Salle F Bruno R Brcic L I Chen-Yost H Jaurand MC . The highlights of the 15th international conference of the international mesothelioma interest group - do molecular concepts challenge the traditional approach to pathological mesothelioma diagnosis? Lung Cancer (2022) 163:1–6. doi: 10.1016/j.lungcan.2021.10.019 34864334

[33] Repo P Staskiewicz A Sutinen E Rönty M Kivelä TT Myllärniemi M . BAP1 germline variants in Finnish patients with malignant mesothelioma. Lung Cancer (2022) 165:102–7. doi: 10.1016/j.lungcan.2022.01.017 35114507

[34] Carbone M Ferris LK Baumann F Napolitano A Lum CA Flores EG . BAP1 cancer syndrome: Malignant mesothelioma, uveal and cutaneous melanoma, and MBAITs. J Transl Med (2012) 10:179. doi: 10.1186/1479-5876-10-179 22935333 PMC3493315

[35] Farley MN Schmidt LS Mester JL Pena-Llopis S Pavia-Jimenez A Christie A . A novel germline mutation in BAP1 predisposes to familial clear-cell renal cell carcinoma. Mol Cancer Res (2013) 11(9):1061–71. doi: 10.1158/1541-7786.MCR-13-0111 PMC4211292 23709298

[36] Wadt KA Aoude LG Johansson P Solinas A Pritchard A Crainic O . A recurrent germline BAP1 mutation and extension of the BAP1 tumor predisposition spectrum to include basal cell carcinoma. Clin Genet (2015) 88(3):267–72. doi: 10.1111/cge.12501 25225168

[37] Jiao Y Pawlik TM Anders RA Selaru FM Streppel MM Lucas DJ . Exome sequencing identifies frequent inactivating mutations in BAP1, ARID1A and PBRM1 in intrahepatic cholangiocarcinomas. Nat Genet (2013) 45(12):1470–3. doi: 10.1038/ng.2813 PMC4013720 24185509

[38] Tang J Xi S Wang G Wang B Yan S Wu Y . Prognostic significance of BRCA1-associated protein 1 in colorectal cancer. Med Oncol (2013) 30(2):541. doi: 10.1007/s12032-013-0541-8 23526420

[39] McDonnell KJ Gallanis GT Heller KA Melas M Idos GE Culver JO . A novel BAP1 mutation is associated with melanocytic neoplasms and thyroid cancer. Cancer Genet (2016) 209(3):75–81. doi: 10.1016/j.cancergen.2015.12.007 26774355 PMC6447287

[40] Petrini I Meltzer PS Kim IK Lucchi M Park KS Fontanini G . A specific missense mutation in GTF2I occurs at high frequency in thymic epithelial tumors. Nat Genet (2014) 46(8):844–9. doi: 10.1038/ng.3016 PMC5705185 24974848

[41] Wadt K Choi J Chung JY Kiilgaard J Heegaard S Drzewiecki KT . A cryptic BAP1 splice mutation in a family with uveal and cutaneous melanoma, and paraganglioma. Pigment Cell Melanoma Res (2012) 25(6):815–8. doi: 10.1111/pcmr.12006 PMC7453745 22889334

[42] Prasad RN Gardner UG Yaney A Prevedello DM Koboldt DC Thomas DL . Germline BAP1 mutation in a family with multi-generational meningioma with rhabdoid features: A case series and literature review. Front Oncol (2021) 11:721712. doi: 10.3389/fonc.2021.721712 34504799 PMC8421801

[43] Laitman Y Newberg J Molho RB Jin DX Friedman E . The spectrum of tumors harboring BAP1 gene alterations. Cancer Genet (2021) 256-257:31–5. doi: 10.1016/j.cancergen.2021.03.007 33866194

[44] Lu C Xie M Wendl MC Wang J McLellan MD Leiserson MD . Patterns and functional implications of rare germline variants across 12 cancer types. Nat Commun (2015) 6:10086. doi: 10.1038/ncomms10086 26689913 PMC4703835

[45] Star P Goodwin A Kapoor R Conway RM Long GV Scolyer RA . Germline BAP1-positive patients: The dilemmas of cancer surveillance and a proposed interdisciplinary consensus monitoring strategy. Eur J Cancer (2018) 92:48–53. doi: 10.1016/j.ejca.2017.12.022 29413689

[46] Rai K Pilarski R Cebulla CM Abdel-Rahman MH . Comprehensive review of BAP1 tumor predisposition syndrome with report of two new cases. Clin Genet (2016) 89(3):285–94. doi: 10.1111/cge.12630 PMC4688243 26096145

[47] Carbone M Pass HI Ak G Alexander HR Jr. Baas P Baumann F . Medical and surgical care of mesothelioma patients and their relatives carrying germline BAP1 mutations(2022) (Accessed 5 May 2022). 10.1016/j.jtho.2022.03.014 PMC9233126 35462085

[48] Walpole S Hayward NK Pritchard AL Johansson PA . Microsimulation model for evaluating the cost-effectiveness of surveillance in *BAP1* pathogenic variant carriers. JCO Clin Cancer Inform (2021) 5:143–54. doi: 10.1200/CCI.20.00124 33513031

[49] McBride E Marlow LAV Forster AS Ridout D Kitchener H Patnick J . Anxiety and distress following receipt of results from routine HPV primary testing in cervical screening: The psychological impact of primary screening (PIPS) study. Int J Cancer (2020) 146(8):2113–21. doi: 10.1002/ijc.32540 PMC7065242 31251820

[50] McBride E Marlow LAV Forster AS Ridout D Kitchener H Patnick J Waller J Anxiety and distress following receipt of results from routine HPV primary testing in cervical screening: The psychological impact of primary screening (PIPS) study. Int J Cancer (2020) 146(8):2113–21. doi: 10.1002/ijc.32540 PMC7065242 31251820

[51] Bell HN Kumar-Sinha C Mannan R Zakalik D Zhang Y Mehra R . Pathogenic ATM and BAP1 germline mutations in a case of early-onset, familial sarcomatoid renal cancer. Cold Spring Harb Mol Case Stud (2022) 8(3):a006203. doi: 10.1101/mcs.a006203 35483881 PMC9059789

